# Temporal and Spatial Variation of Radon Concentrations in Environmental Water from Okinawa Island, Southwestern Part of Japan

**DOI:** 10.3390/ijerph18030998

**Published:** 2021-01-23

**Authors:** Shunya Nakasone, Akinobu Ishimine, Shuhei Shiroma, Natsumi Masuda, Kaori Nakamura, Yoshitaka Shiroma, Sohei Ooka, Masahiro Tanaka, Akemi Kato, Masahiro Hosoda, Naofumi Akata, Yumi Yasuoka, Masahide Furukawa

**Affiliations:** 1 Graduate School of Engineering and Science, University of the Ryukyus, Nishihara, Okinawa 903–0213, Japan; k198345@eve.u-ryukyu.ac.jp (K.N.); m_furu@sci.u-ryukyu.ac.jp (M.F.); 2 Department of Radioecology, Institute for Environmental Sciences, Rokkasho, Aomori 039–3212, Japan; aki_ishi@ies.or.jp; 3 Faculty of Science, University of the Ryukyus, Nishihara, Okinawa 903–0213, Japan; shiromashuhei1101@gmail.com (S.S.); 83ka.ha.ho@gmail.com (N.M.); 4 Faculty of Education, University of the Ryukyus, Nishihara, Okinawa 903–0213, Japan; y_shiro@cs.u-ryukyu.ac.jp; 5 Nanto Co., Ltd., Naha, Okinawa 900–0013, Japan; oooka@gyokusendo.co.jp; 6 Department of Helical Plasma Research, National Institute for Fusion Science, National Institutes of Natural Sciences, Toki, Gifu 509–5292, Japan; tanaka.masahiro@nifs.ac.jp; 7 School of Physical Sciences, The Graduate University for Advanced Studies, SOKENDAI, Toki, Gifu 509–5292, Japan; 8 Department of Engineering and Technical Services, National Institute for Fusion Science, National Institutes of Natural Sciences, Toki, Gifu 509–5292, Japan; kakemi@nifs.ac.jp; 9 Department of Radiation Science, Hirosaki University Graduate School of Health Sciences, Hirosaki, Aomori 036–8564, Japan; m_hosoda@hirosaki-u.ac.jp; 10 Department of Radiation Chemistry, Institute of Radiation Emergency Medicine, Hirosaki University, Hirosaki, Aomori 036–8564, Japan; akata@hirosaki-u.ac.jp; 11 Radioisotope Research Center, Kobe Pharmaceutical University, Kobe, Hyogo 658–8558, Japan; yasuoka@kobepharma-u.ac.jp

**Keywords:** radon concentration, groundwater, residence time, limestone aquifer, Okinawa Island

## Abstract

In this study, to get a better understanding in characterizing groundwater and ensure its effective management, the radon concentrations in water samples were measured through Ryukyu limestone in southern Okinawa Island, Japan. Water samples were collected from a limestone cave (Gyokusendo cave, dropping water) and two springs (Ukinju and Komesu, spring water), and the radon concentrations were measured by liquid scintillation counters. The radon concentrations in the samples from the Gyokusendo cave, and Ukinju and Komesu springs were 10 ± 1.3 Bq L^−1^, 3.2 ± 1.0 Bq L^−1^, and 3.1 ± 1.1 Bq L^−1^, respectively. The radon concentrations showed a gradually increasing trend from summer to autumn and decreased during winter. The variation of radon concentrations in the dripping water sample from the Gyokusendo cave showed a lagged response to precipitation changes by approximately 2–3 months. The estimated radon concentrations in the dripping water sample were calculated with the measured radon concentrations from the dripping water obtained during the study period. Based on our results, groundwater in the Gyokusendo cave system was estimated to percolate through the Ryukyu limestone in 7–10 days, and the residence time of groundwater in the soil above Gyokusendo cave was estimated to be approximately 50–80 days. This work makes a valuable contribution to the understanding of groundwater processes in limestone aquifers, which is essential for ensuring groundwater sustainability.

## 1. Introduction

Radon is a radioactive noble gas with an atomic number of 86. Although there are many isotopes of radon, only three occur in the natural environment: ^222^Rn, ^220^Rn (Thoron), and ^219^Rn (Actinon), with half-lives of 3.8 d, 55.6 s, and 3.9 s, respectively. ^222^Rn is the most frequently used radon isotope due to its relatively long half-life compared to thoron and actinon. Hereinafter, “radon” refers to ^222^Rn. Radon is a natural radionuclide belonging to the uranium (^238^U) decay series, with radium (^226^Ra) as a parent nuclide. The ^238^U decay series ends with ^206^Pb, a stable isotope of lead. Inhalation of radon and its decay products is believed to increase the risk of lung cancer [1]. Most radon in the environment is produced by the alpha decay of radium in the soil and rocks. Since radon is extremely soluble in water (e.g., 0.295 cm^3^ kg^−1^ at 15 °C), there may be an exchange and equilibrium between pore-water and pore-gas when the soil is unsaturated [2]. In addition, radon is chemically inert. Therefore, if it is dissipated from soil grains into pore water or voids, it can migrate relatively far from where it is produced by advection or diffusion [2]. Eventually, some of the radon dissipated in the pore water migrates to the groundwater and is dissolved in spring or hot spring water. Radon, which is widely distributed in the natural environment, is commonly used to study atmospheric transport processes and groundwater flow systems such as groundwater recharge, flow, and discharge [3,4].

Radon can be used as a hydrological tracer for estimating the residence time of groundwater under very special boundary conditions (e.g., in limestone areas or for submarine groundwater discharge in the coastal zone) [5,6,7]. In karst aquifers, researchers have analyzed radon concentrations to understand the recharge dynamics of water passing through the soil layer to the saturated zone [8]. Additionally, groundwater and soil radon concentrations are measured during irrigated and non-irrigated periods in paddy fields, and the difference in the concentration values between groundwater and surface water is used to evaluate surface water infiltration into the aquifer [9]. Radon can also be used for estimating the probability of geophysical risk events such as volcanic activity and earthquakes [10,11,12]. Pre-earthquake radon anomalies have been found in soil gases, groundwater, and spring water [13,14]. Radon serves as a useful geochemical tracer for studying the short-term environmental characteristics of cave systems, such as the flow of the atmospheric circulation. Variations in the air density and atmospheric radon concentrations inside and outside caves have been reported for the seasonal natural ventilation in the cave [15,16,17].

The storage capacity of a limestone layer alone is low due to it being highly permeable and porous. However, when a limestone layer overlies an impermeable layer in a non-conformant way, as is the case on Okinawa Island, the limestone serves as an aquifer. Limestones can serve as excellent natural aquifers and provide drinking water to 25% of the world’s population [18]. Moreover, water in limestone aquifers is the only available drinking water in some regions and serves as a valuable water source for agriculture [18].

Okinawa Island, a Japanese island in the East China Sea, is characterized by short and steep river channels. Moreover, because its watershed area is small, the rainfall is discharged directly into the ocean. Okinawa Island experienced a relatively high annual precipitation of 2040 mm during the 1981–2010 period [19]. However, due to significant seasonal and interannual changes, the flow of rivers on the island is unstable and the amount of water available is limited. Therefore, it is difficult to secure stable water resources in Okinawa Island from river water alone and part of the water for agriculture and domestic use is provided by groundwater. To establish a stable supply and ensure the sustainable use of groundwater in the future, it is necessary to determine the groundwater characteristics, such as availability and residence time, in limestone aquifers. However, because of the heterogeneous permeability and complex hydrogeological structure of limestone, it is difficult to understand the behavior of groundwater from existing numerical models. For example, groundwater infiltration and residence time in limestone aquifers vary with each precipitation event.

Characteristics of local groundwater processes can be inferred from radon measurements. Shiroma et al. (2016) intermittently measured the radon concentration in dripping water exuding from the ceiling of a cave and reported that it is a potential source of radon in the cave atmosphere [20]. Although other factors may be involved, by comparing radon concentrations and monthly precipitation, changes in radon concentrations were shown to correspond to precipitation changes after a delay of 60–90 days. Furthermore, groundwater was calculated to percolate through the Ryukyu limestone in 9 to 10 days. However, the radon concentration data were collected intermittently for only one year and only at one site.

The main objective of this study is as attempt to calculate the residence time of groundwater in limestone areas. The study area comprised a limestone cave and two springs in the southern part of Okinawa Island. The residence time of groundwater from the soil to the cave was calculated based on the relationship between the radon concentration of the dripping water from the cave ceiling and the monthly precipitation. Moreover, we sampled two springs in the study area to understand the spatial distribution of radon concentrations in the groundwater.

## 2. Materials and Methods

### 2.1. Overview of the Study Site

**Figure 1:**
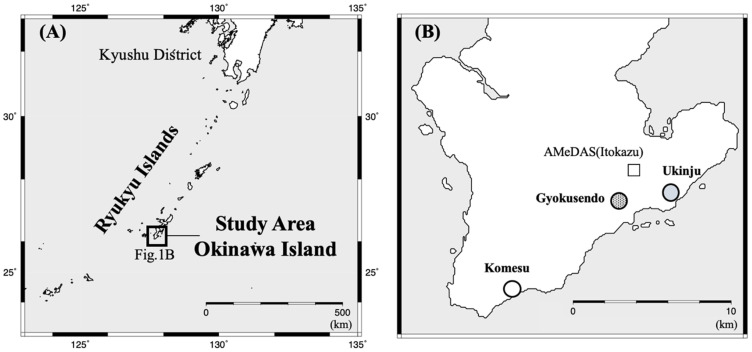
Locations of the study area (**A**) and sampling sites (**B**). The white square shows the Automated Meteorological Data Acquisition System (AMeDAS) situated at Itokazu.

Okinawa Prefecture, located in southwestern Japan, is the only prefecture in the country with a subtropical climate (Figure 1A). Ryukyu limestone, which originates from coral reef sediments, formed approximately one million to 500,000 years ago [21]. Limestone is widely distributed, and there are numerous limestone caves in Okinawa. The southern part of Okinawa Island consists of the Neogene-Quaternary Shimajiri Group and the Ryukyu Group, with low-lying terrain below 200 m in elevation. The Shimajiri Group of the Neogene Pliocene is predominantly composed of mudstone and is prone to erosion, while the limestone Quaternary Pleistocene Ryukyu Group, which is predominantly composed of limestone, is more resistant to erosion and forms a flat-surfaced topography [22]. The Ryukyu limestone overlies the Shimajiri Group in a non-conformant way. The Ryukyu limestone is permeable, while the Shimajiri Group is impermeable [23]. Therefore, Ryukyu limestones serve as aquifers in Okinawa Island (i.e., Ryukyu aquifer).

Okinawa Island has three major soil types: Shimajiri Mahji, Kunigami Mahji, and Jahgaru. Shimajiri Mahji is a dark red soil, which is widely distributed in the Ryukyu limestone distribution area [24], with a reddish brown to dark brown color that is neutral to slightly alkaline [25]. Dark red soil is derived from the weathering products of limestone and/or a high-background radiation area, mainly the southeastern part of China [25].

### 2.2. Sample Collection

Water samples were collected from Gyokusendo cave (26°08′25″ N, 127°44′57″ W, 46 m altitude, dripping water), Ukinju spring (26°08′15″ N, 127°47′35″ W, 6.5 m altitude, spring), and Komesu spring (26°05′18″ N, 127°42′04″ W, 3.2 m altitude, coastal spring), all located in the southern portion of Okinawa Island (Figure 1B). Water samples were collected from October 2016 to October 2020. Sample collection at Ukinju and Komesu started in April 2018 and May 2018, respectively. The collection period in the springs was delayed because it took time to find a location with a high flow rate in the coastal area. However, data for April to June 2020 are missing for each location due to the COVID-19 pandemic.

**Figure 2:**
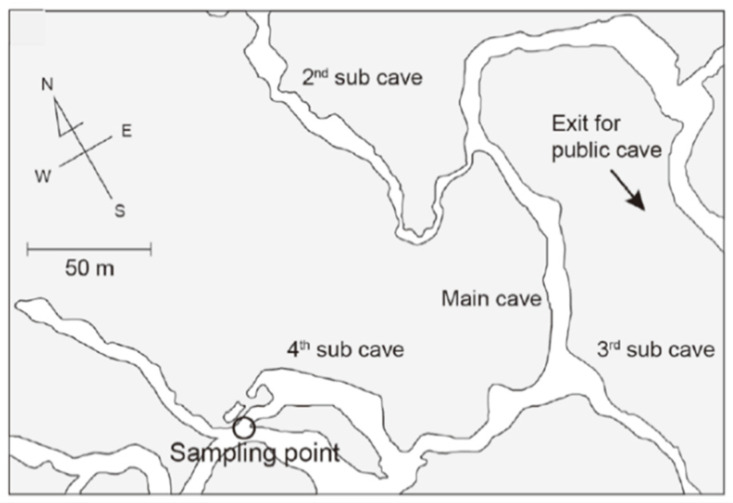
Location of the sampling point in Gyokusendo cave (map from, Shiroma et al. 2016 [20]).

Gyokusendo cave is a limestone cave situated in a 120-m-thick body of Ryukyu limestone. Shimajiri Mahji soil is distributed on the surrounding ground surface. The total length of Gyokusendo cave reaches 5000 m, and approximately 800 m are open for tourism [26]. A concrete staircase with a height of approximately 30 m serves as an entrance to the main cave. A branch cave in an undisclosed area about 30 m away from the stalagmite named “Shoryu no Kane” was selected as the water sampling point (Figure 2). There are soda-straw stalactites (straws) at this site with a significantly high drip rate. Therefore, it was possible to collect 10 mL of dripping water in approximately 10 min. The Ryukyu limestone is exposed at Ukinju spring and Komesu spring, and Shimajiri Mahji is distributed in the surrounding ground surface at both sites. The Ukinju and Komesu springs are located near the coast, 170 m and 12 m from the coastline, respectively. 

For sample collection, 20 mL high-performance glass vials (PerkinElmer, USA) containing 10 mL of high efficiency mineral oil scintillator (PerkinElmer, USA) were prepared. For each site, 5 samples were collected. The water samples were collected using a syringe with the needle removed. Water samples (12 mL) were collected directly from the straw formed in the cave ceiling in Gyokusendo cave. At the Ukinju and Komesu sites, the same sample amounts were collected near the spring outlets. The syringe was pointed upward, and the plunger was pressed carefully to remove air from the inside. A needle was then attached, and the tip of the needle was positioned just over the bottom of the glass vial, and 10 mL of sample was injected. The time elapsed from water sample injection to the closing of the lid of the glass vial was set as the water sampling time. After water sampling, the samples were shaken for 60 s and left in the dark for 4 h until radiative equilibrium was achieved to extract the radon from the sample water into the mineral oil scintillator.

Temperature and humidity data in the cave were measured using HOBO Pro v2 Loggers (U23–001, ONSET, USA). However, data for December 2016 and February 2017 were missing due equipment failure. Precipitation data outside the cave were obtained from the Automated Meteorological Data Acquisition System (AMeDAS) situated at Itokazu (Figure 1B). The pH and electrical conductivity (EC) of the water samples were measured using a pH meter (AS-712pH, HORIBA) and an EC meter (B-771COND, HORIBA). To verify the existence of saltwater inflow, the salinity (PSU; Practical Salinity Unit) was also measured at Ukinju and Komesu spring located in the coastal area using a digital salinity meter (HI96822, HANNA instruments).

### 2.3. Analysis of Radon Concentration in Water

The radon concentrations in water were measured for 60 min per sample using a liquid scintillation counter. The measurements were performed using a Quantulus 1220 (PerkinElmer, USA), at the National Institute for Fusion Science, from October 2016 to February 2019 and a Tri-Carb 2019TR (PerkinElmer, USA), at the University of the Ryukyus Center for Research Advancement and Collaboration, from March 2019 to October 2020. The radon concentration $C_{R}$ (Bq L^−1^) was calculated using the following equation:

(1) $$C_{R}=\frac{\left(A_{0}-B_{0}\right)\exp\left(\lambda T_{p}\right)\;\;}{f_{\mathrm{Rn}}V}$$

where $A_{0}$ is the counting rate (cps) of the water sample, $B_{0}$ is the counting rate (cps) of the background sample, *λ* is the radon decay constant (day^−1^), $T_{p}$ is the elapsed period (day), which was corrected for radioactive decay to between the sampling time and the middle measurement time, $f_{\mathrm{Rn}}$ is the radon conversion factor of 3.24 cps Bq L^−1^ for the Quantulus 1220 and 4.5 cps Bq^−1^ for the Tri-Carb2019TR, and *V* is the sample volume (0.01 L) [27]. 

For measurements using the Quantulus 1220, $A_{0}$ and $B_{0}$ were calculated using the counting rate cps for 600–910 ch. Additionally, for measurements using the Tri-Carb2019TR, $A_{0}$ and $B_{0}$ were calculated using the counting rate cps for 0–1024 ch using the integral counting method [27,28,29]. The minimum detected radon concentration *MDC* (Bq L^−1^) was calculated using the following equation [30]:

(2) $$N_{D}=\;4.65\;\;\sqrt{\frac{B_{0}}{t}}+\frac{2.71}{t}$$

(3) $$MDC\;=\;\frac{N_{D}}{f_{Rn}\;V\;}$$

where $N_{D}$ is the minimum value of net count rate (cps), t is the counting time of the sample and the background (sec). The minimum detected radon concentrations by this method were 1.0 Bq L^−1^ for the Quantulus1220 and 0.9 Bq L^−1^ for the Tri-Carb2019TR [30].

Uncertainties were estimated for the following components: the counting rate (cps) of the water sample and background sample, the measurement time (s) of the water sample and background sample, the radon decay constant (s^−1^), the elapsed period (s), the radon conversion factor (cps Bq L^−1^), and the sample volume (L). Estimates were also made for each of these components, which were combined to give the expanded uncertainty [31]. By applying a coverage factor of *k =* 2, the expanded uncertainty was evaluated as 10%. In this study, radon concentrations are expressed as the arithmetic mean ± standard deviation of 5 samples.

## 3. Results and Discussion

### 3.1. Physical Parameters and Meteorological Data

**Figure 3:**
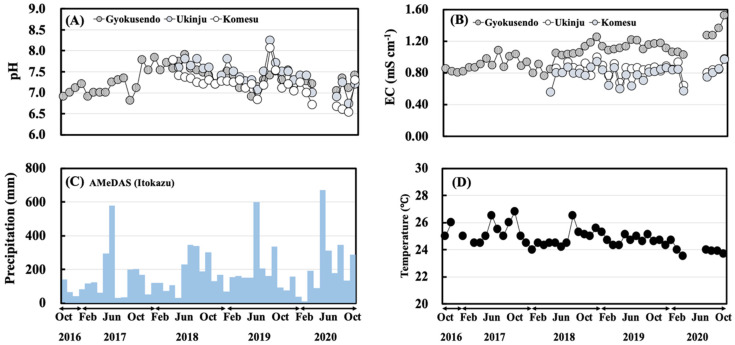
Monthly variations for the October 2016–October 2020 period of pH (**A**), electrical conductivity (**B**), and precipitation (**C**) in the water samples, and temperature (**D**) in the Gyokusendo cave.

The pH and EC of the water samples and the monthly precipitation data are shown in Figure 3. The pH and EC range of the samples from the Gyokusendo cave were 6.8–7.9, 0.76–1.52 mS cm^−1^. For the Ukinju spring, the pH and EC ranged from 6.7–8.2 and 0.64 and 0.99 mS cm^−1^. For the Komesu spring, the pH ranged between 6.5–8.1 and the EC measured between 0.55 and 0.96 mS cm^−1^.

The results reveal no significant seasonal variation in pH and EC. The monthly precipitation ranged from 11.50–669.50 mm. The monthly precipitation tended to be higher during the rainy season (May-June) and the typhoon season (August-October) and lower in winter (December–February). The salinity of Ukinju spring and Komesu spring, located near the coast, was in the range of 0–1 PSU, and seawater contamination was not observed. The temperature inside the Gyokusendo cave is shown in Figure 3D. The temperature outside the cave showed a marked seasonal variation (high in summer, low in winter) of 15–27 °C [19], while inside the cave, the temperature varied marginally, ranging from 23–27 °C. The humidity in the cave was generally 100% throughout the measurement period.

### 3.2. Temporal Variation of Radon Concentrations for the Water Samples

**Figure 4:**
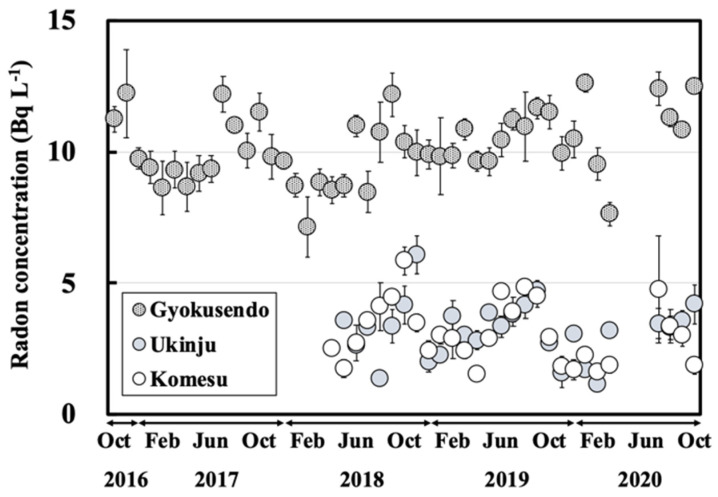
Radon concentration variation for the Gyokusendo cave, Ukinju spring and Komesu spring water samples collected during the October 2016-October 2020 sampling period.

Radon concentrations for the dripping water collected at the Gyokusendo cave ranged from 7.1–13 Bq L^−1^ (Figure 4), with an average of 10 ± 1.3 Bq L^−1^. The radon concentration for the dripping water collected at the same site between May 2013 and March 2014 was relatively consistent and ranged between 6.4 to 15 Bq L^−1^ [20]. The radon concentration for the dripping water increased gradually from summer (June-August) to autumn (September-November) and then decreased in winter (December-February).

**Figure 5:**
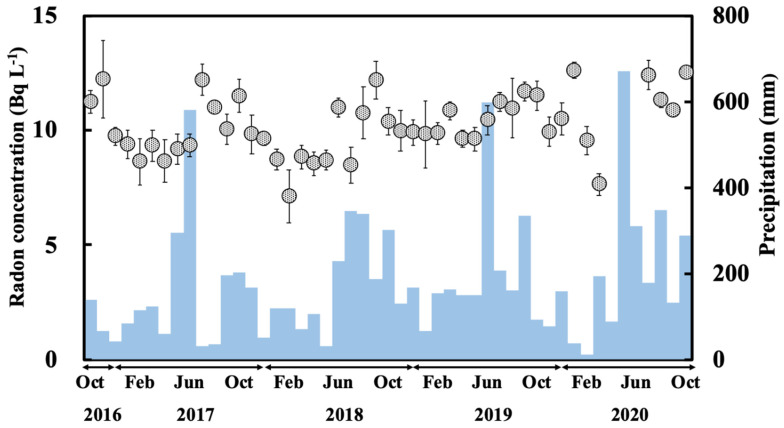
Variation of radon concentrations for the dripping water sample from the Gyokusendo cave compared to the monthly precipitation obtained from AMeDAS (Itokazu) for the study period.

The monthly average radon concentrations of the dripping water for the entire sampling period were the highest (11 ± 0.8 Bq L^−1^) in October and the lowest (8.8 ± 1.2 Bq L^−1^) in February (Figure 5). These variations were similar to the seasonal variations in atmospheric radon concentrations in the Gyokusendo cave sample (high in summer and low in winter) observed between July 1990 and January 1993 [15,16,17]. The radon concentrations for the water collected at Ukinju spring and Komesu spring were 3.2 ± 1.0 Bq L^−1^ (1.1–6.1 Bq L^−1^) and 3.1 ± 1.1 Bq L^−1^ (1.1–5.8 Bq L^−1^) (Figure 4). There was a gradual increase in radon concentrations from summer to autumn. However, no clear seasonal variation was apparent (Figure 4).

### 3.3. Relationship between Radon Concentration for Dripping Water and Monthly Precipitation

The temporal variation in the radon concentrations of the dripping water from the Gyokusendo cave and the precipitation recorded for the study period are shown in Figure 5. The monthly precipitation was approximately 500 mm during the rainy season, increased during the typhoon season, and decreased to approximately 100–150 mm during winter.

In May 2017, an anomalously high precipitation amount of 579 mm (equivalent to approximately 30% of the 2017 annual precipitation) was recorded, and the radon concentration of the dripping water in July of that year was 12 Bq L^−1^, the highest recorded in 2017. The precipitation from May to June 2018 was lower than usual, averaging a monthly amount of 300 mm. However, in September 2018, the radon concentration in the dripping water from the cave was exceptionally high, reaching 12 Bq L^−1^. In 2019, the highest amount of precipitation for the year (597 mm) was recorded in June, and the maximum radon concentration for the year was recorded in September (12 Bq L^−1^). More than 600 mm of precipitation was observed in June 2020, and maximum radon concentrations of 12 Bq L^−1^ and 13 Bq L^−1^ were recorded in July and October 2020. Conversely, a decreasing precipitation trend corresponded to a decrease in the radon concentration. Additionally, time series analysis (moving average) of the radon concentrations in dripping water and precipitation showed a correlation between the 2-month moving average (*R* = 0.50) and the 3-month moving average (*R* = 0.50) [32]. Therefore, variations in radon concentrations of the dripping water samples indicated that the radon concentration increased during periods of heavy rainfall, although a delay of around two to three months after a monthly precipitation change was observed. This suggests that precipitation percolates at a rate of 60 to 90 d from the soil to the Ryukyu limestone situated above the Gyokusendo cave and reaches the cave as dripping water. 

Radon concentrations in groundwater reflect the inherent ^226^Ra contents of the host rock or formation [33]. In areas where karst aquifers are distributed, estimated radon concentrations in the soil are higher than the radon concentrations in the groundwater. Therefore, the main source of radon in groundwater has been reported to be soil [8]. Furthermore, the concentrations of the ^238^U series in the Ryukyu limestone (8.6 Bq kg^−1^) were reported to be lower than those in Shimajiri red soil (86.0 Bq kg^−1^) [34], suggesting that the main source of radon in groundwater is the soil deposited in the upper part of the Gyokusendo cave. Additionally, the amount of soil water infiltrating into the aquifer increases with heavy precipitation [7]. In limestone areas, the residence time of soil water was noted to reflect the residence time in the limestone bodies [8]. The flow velocity is proportional to the flow rate and inversely proportional to the cross-sectional area of the channel. The flow velocity is the distance an object moves per unit time, the flow rate is the volume of fluid per unit time flowing past a point through the cross-sectional area. The channel is referring to solution openings through the limestone. The following equations was used to calculate the flow velocity:

(4) $$\nu =\;\frac{Q}{A}$$

where ν is the flow velocity (m s^−1^), Q is the flow rate (m^3^ s^−1^), and A is the cross-sectional area of the channel (m^2^). It is assumed that the main source of radon in groundwater is the soil deposited in the upper part of the Gyokusendo cave and that the cross-sectional area of the channel does not change significantly. Therefore, radon in the dripping water is derived from radon in the soil pore water, which is governed by the soil ^226^Ra content and the residence time of groundwater in the Ryukyu limestone. Moreover, the amount of groundwater in the Ryukyu limestone depends on precipitation. High precipitation rates shorten the residence time of groundwater in the Ryukyu limestone by increasing infiltration into the soil, which accelerates the groundwater flow velocity. Low precipitation rates reduce infiltration into the soil, thus, decreasing the groundwater flow velocity and increasing the residence time of groundwater. Attenuation is constant (the half-life, 3.8 day) over time. Therefore, the longer the residence time, the lower the radon concentration and vice versa. In this study, the radon concentration trends correlated with the precipitation trends, suggesting that the soil may be the source of radon in the groundwater. Moreover, the variation in the radon concentration of the dripping water sample may have been due to the residence time of groundwater in the Ryukyu limestone.

### 3.4. Estimation of Residence Time Using Radon Decay in the Dripping Water

**Figure 6:**
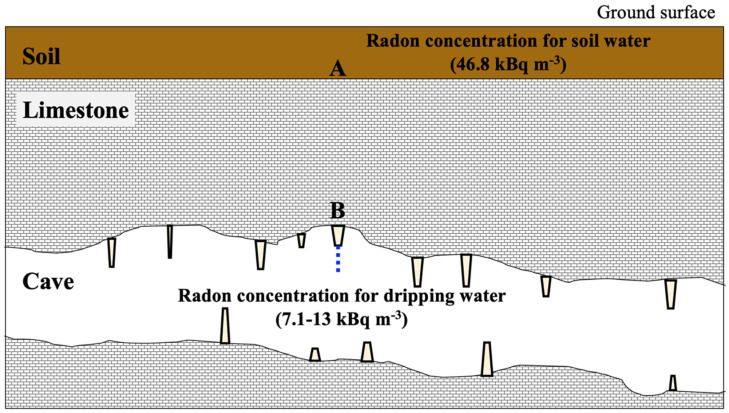
Schematic of the Gyokusendo cave profile, where A is the boundary between the soil and the limestone, and B is the target straw stalactite.

A schematic of a vertical section of the Gyokusendo cave is shown in Figure 6. In the figure, A shows the boundary between the soil and the Ryukyu limestone, and B is a straw stalactite on the cave ceiling where the dripping water is collected. Precipitation percolates through the soil and limestone bodies above the cave and seeps into the cave from the ceiling as dripping water. Since radon is rarely present in precipitation, radon in groundwater is mainly supplied by the soil. The concentration of radon in water infiltrated into the ground increases with time due to the supply of radon produced by soil particles. However, as the concentration increases, the number of decaying radon atoms increases, and eventually, an equilibrium state is reached where supply and decay are equal. Therefore, radon in the soil water and ^226^Ra in the solid phase are in permanent equilibrium. Additionally, if radon remains in the soil long enough, the concentration of radon in the gas and liquid phases will reach the equilibrium state specific to each soil species. The following equations were used to quantify the radon concentration in soil water and the gas phase at gas-liquid equilibrium:

(5) $$E_{w}=K_{T}\;E_{a}$$

where $E_{w}$ is the radon concentration in soil water (kBq m^−3^), $K_{T}$ is the radon partition coefficient, and $E_{a}$ is the radon concentration in the gas phase (kBq m^−3^). The radon partition coefficient was calculated using the following equation [35]:

(6) $$K_{T}=\frac{9.12}{17.0+T}\;$$

where *T* is the temperature (°C), and the mean value of the temperature in the cave, 24.9 °C, was used.

According to the UNSCEAR 2000 (United Nation Scientific Committee on the Effects of Atomic Radiation 2000), the concentration of radon in soil pore gas $E_{a}$ (Bq m^−3^) can be calculated using the following equation [36]:

(7) $$E_{a}=\;C_{\mathrm{Ra}}\;f\;\rho_{s}\;\varepsilon^{-1}\;\left(1-\varepsilon \right)\;{\left[m\left(K_{T}-1\right)+1\right]}^{-1}$$

where $C_{\mathrm{Ra}}$ is the concentration of ^226^Ra in the soil (Bq kg^−1^), *f* is the radon emanation coefficient, $\rho_{s}$ is the density of solid particles (kg m^−3^), ε is the porosity, and *m* is the water saturation. In this study, based on a previous report [37], the radon emanation coefficient for typical soil on Okinawa Island was set to 0.40, the concentration of ^226^Ra in soil was set to 106 Bq kg^−1^ dry, the solid particle density was set to 2697 kg m^−3^, and the porosity was set to 0.67. The water saturation was 0.95, which is representative of the UNSCEAR 2000 [36]. The radon partition coefficient $K_{T}$ was calculated using Equation (6) [35]. Since we could not directly measure the soil temperature, we used the temperature in the cave (24.9 °C) as approximation of the soil temperature. Radon concentrations (Bq m^−3^) in soil water were calculated using Equation (5). The above values were defined as the radon concentration in the groundwater at point A (Figure 6).

Because radon was not supplied from the Ryukyu limestone, we assumed that the radon concentration in the groundwater was attenuated by the residence time of the groundwater in the Ryukyu limestone. The estimated radon concentration in the dripping water sample $E_{d}$, exuding from point B (Figure 6), was calculated using the estimated radon concentration in soil water from Equation (5) and the following equation.

(8) $$E_{d}=E_{w}\exp\left(-\lambda t\right)$$

where *t* is the residence time (days) of groundwater in the Ryukyu limestone.

The estimated radon concentrations in the dripping water sample calculated from Equation (8) were compared with the measured radon concentrations in the dripping water obtained during the study period. The radon concentration in the soil pore gas calculated from Equation (7) was 215 kBq m^−3^. The radon concentration in the soil pore water calculated from Equation (5) was 46.8 kBq m^−3^. Radon concentrations for the dripping water sample obtained during the study period ranged from 7.1–13 kBq m^−3^ as shown in Figure 4. Based on the decay of radon (half-life: 3.8 d) from point A (estimated values) to point B (measured values) and the assumption that the residence time of soil and Ryukyu limestone is 60 to 90 d, the residence time of groundwater in the Ryukyu limestone was estimated to be 7 to 10 d. These results are generally consistent with previously reported values (9–10 d) [20]. Moreover, the residence time of groundwater in the soil deposited in the upper part of the Gyokusendo cave was estimated to be approximately 50 to 80 d.

The residence time of groundwater in the Ryukyu limestone at the Ukinju spring and Komesu spring sites was also calculated from Equation (8). For this calculation, we assumed that no radon was supplied from the Ryukyu limestone, the soil was uniformly deposited, and the radon concentration in the soil was in radiative equilibrium. The radon concentrations for the water samples from the Ukinju spring and Komesu spring sites ranged from 3.1–6.1 Bq L^−1^ and 1.1–5.8 Bq L^−1^, respectively. Based on the radon attenuation between the measured radon concentrations in the water samples and the estimated radon concentrations in the pore water, the residence times in the Ryukyu limestone at Ukinju spring and Komesu spring were estimated to be 12 to 21 d and 12 to 19 d, respectively. Nakaya et al. (2018) reported an average residence time in the Ryukyu limestone aquifer of 14–34 years based on the results of SF_6_ concentrations in the spring and well water around the Komesu spring site [38]. The residence time estimated in this study is very limited because it was calculated based on observations at only one point in the catchment. Although we were able to calculate the residence time, our results significantly differ from those of previous studies. In the future, it will be necessary to discuss the residence time after considering additional factors such as the frequency of collection, and number of sites, and depth of sampling sites.

## 4. Conclusions

Radon concentrations for dripping water and spring water samples were collected in the southern part of Okinawa Island from October 2016 to October 2020. The estimated radon concentrations in the dripping water sample were calculated with the measured radon concentrations from the dripping water obtained during the study period. Based on our results, we determined the following:

(1) The radon concentrations in the water samples from the Gyokusendo cave, Ukinju spring, and Komesu spring were 10 ± 1.3 Bq L^−1^, 3.2 ± 1.0 Bq L^−1^, and 3.1 ± 1.1 Bq L^−1^, respectively. Radon concentrations for the water samples showed a gradually increasing trend from summer to autumn and decreased in the winter. This was particularly noticeable in the radon concentrations measured in the dripping water sample from the Gyokusendo cave.
(2) From the variation in the radon concentrations for the dripping water and precipitation, we estimated that the radon concentration changes in the dripping water lags precipitation changes by approximately 2–3 months. These results indicate that precipitation takes 60 to 90 d to percolate into the soil and accumulate in the Ryukyu limestone overlying the Gyokusendo cave. The water then enters the cave as dripping water from the cave ceiling.
(3) From a simple radon behavior model, groundwater in the Gyokusendo cave system was estimated to percolate through the Ryukyu limestone in 7 to 10 d, and the residence time of groundwater in the soil above the Gyokusendo cave was estimated at approximately 50 to 80 d.
(4) The residence times of groundwater in the Ryukyu limestone at the Ukinju spring and Komesu spring sites were calculated to be 12 to 21 d and 12 to 19 d, respectively. However, this estimation was lower than that of previous studies (14–34 years). Therefore, it will be necessary for future studies to discuss the residence time after understanding factors such as the frequency of collection, and the number of sites, and depth of sampling sites. Additionally, to verify the residence time and mixing of groundwater in the area, radioactive isotopes with longer half-lives, such as tritium (half-life: 12.3 y) should be analyzed in the future.

## References

[1] World Health Organization (2009). WHO Handbook on Indoor Radon: A Public Health Perspective.

[2] Nazaroff W.W. (1992). Radon transport from soil to air. Rev. Geophys..

[3] Hirao S., Yamazawa H., Moriizumi J., Yoshioka K., Iida T. (2008). Development and verification of long-range atmospheric radon-222 transport model. J. Nucl. Sci. Technol..

[4] Csondor K., Erőss A., Horváth Á., Szieberth D. (2017). Radon as a natural tracer for underwater cave exploration. J. Environ. Radioact..

[5] Hoehn E., Von Guten H.R. (1989). Radon in groundwater: A tool to assess infiltration from surface waters to aquifers. Water Resour. Res..

[6] Ellins K.K., Roman-mas A., Lee R. (1990). Using ^222^Rn to examine groundwater/surface discharge interaction in the Rio Grande de Manati, Puerto Rico. J. Hydrol..

[7] Hamada H. (2000). Estimation of groundwater flow rate using the decay of ^222^Rn in a well. J. Environ. Radioact..

[8] Savoy L., Subeck H., Hunkeler D. (2011). Radon and CO_2_ as natural tracers to investigate the recharge dynamics of karst aquifers. J. Hydrol..

[9] Hamada H., Komae T. (1998). Analysis of recharge by paddy field irrigation using ^222^Rn concentration in groundwater as an indicator. J. Hydrol..

[10] Igarashi G., Saeki S., Takahata N., Sumikawa K., Tasaka S., Sasaki Y., Takahashi M., Sano Y. (1995). Ground-water radon anomaly before the Kobe earthquake in Japan. Science.

[11] Yasuoka Y., Shinogi M. (1997). Anomaly in atmospheric radon concentration: A possible precursor of the 1995 Kobe, Japan, earthquake. Health Phys..

[12] Koike K., Yoshinaga T., Asaue H. (2014). Characterizing long-term radon concentration changes in a geothermal area for correlation with volcanic earthquakes and reservoir temperatures: A case study from Mt. Aso, southwestern Japan. J. Volcanol. Geotherm. Res..

[13] Oh H.Y., Kim G. (2015). A radon-thoron isotope pair as a reliable earthquake precursor. Sci. Rep..

[14] Kuo T., Fan K., Kuochen H., Han Y., Chu H., Lee Y. (2006). Anomalous decrease in groundwater radon before the Taiwan M6.8 Chengkung earthquake. J. Environ. Radioact..

[15] Tanahara A., Taira H., Takemura M. (1997). Radon distribution and the ventilation of a limestone cave on Okinawa. Geochem. J..

[16] Tanahara A., Iha H., Taira H. (1997). Factors controlling the changes in ^222^Rn concentration in Gyokusendo cave on Okinawa Island. J. Speleol. Soc. Jpn..

[17] Iha H., Tanahara A., Taira H. (1999). A critical analysis of changing radon concentration patterns on Gyokusendo cave in Okinawa Island. J. Speleol. Soc. Jpn..

[18] Ford D.C., Williams P.W. (1989). Karst Hydrogeology and Geomorphology.

[19] Japan Meteorological Agency Automated Meteorological Data Acquistion System (AMeDAS). https://www.data.jma.go.jp/obd/stats/etrn/index.php.

[20] Shiroma Y., Shiroma M., Kina S., Hosoda M., Yasuoka Y., Akata N., Furukawa M. (2016). Source of atmospheric radon in the Gyokusendo, a limestone cave in Okinawa, Japan. Jpn. J. Health Phys..

[21] Iryu Y., Matsuda H., Machiyama H., Piller E.W., Quinn M.T., Mutti M. (2006). Introductory perspective on the COREF project. Island Arc.

[22] Furukawa H., Kizaki K. (1985). The southern part of Okinawa Island. Geological Feature of the Ryukyu Arc.

[23] Kaneko N., Ujiie H., Kaneko N. (2006). Ryukyu Group. Geology of the Itoman and Kudaka Jima District.

[24] Hamazaki T. (1979). Parent materials and soils of Nansei-shoto in Japan. Pedologist.

[25] Tokashiki Y. (1993). The characteristic properties of the Shimajiri Mahji and Jahgaru soils in Okinawa prefecture. Pedologist.

[26] Ishihara Y., Yoshimura K., Ooka S., Sasaki H., Yamazaki S. (2018). Topography, geology of Sakitarido ruins and ruins formation process. Excavation Report of the Sakitari-Do Cave Site, Nanjo City, Okinawa Pref..

[27] Tanaka R., Araki S., Yasuoka Y., Mukai T., Ohnuma S., Ishikawa T., Fukuhori N., Sanada T. (2013). A simplified method for improved determination of radon concentration in environmental water samples. Radioisotopes.

[28] Yasuoka Y., Ishii T., Kataoka Y., Kubo T., Suda H., Tokonami S., Ishikawa T., Shinogi M. (2004). Determination of radon concentration in water using liquid scintillation counter. Radioisotopes.

[29] Yasuoka Y., Ishikawa T., Fukuhori N., Tokonami S. (2009). Comparison of simplified liquid scintillation counter (Triathler) with conventional liquid scintillation counter in the measurement of radon concentration in water. J. Hot Spring Sci..

[30] Knoll G.F. (2000). Radiation Detection and Measurement.

[31] Kraguten J. (1994). Tutorial review. Calculating standard deviations and confidence intervals with a universally applicable spread sheet technique. Analyst.

[32] Shumway R.H., Stoffer D.S. (2017). Time Series Analysis and Its Applications with R Examples.

[33] Tenner A.B., Actams J.A.S., Lowder W.M. (1964). Physical and chemical controls on the distribution of radium-226 and radon-222 in groundwater near Great Salt Lake, Utah. The Natural Radiation Environment.

[34] Furukawa M., Kina S., Shiroma M., Shiroma Y., Masuda N., Motomura D., Hiraoka H., Fujioka S., Kawakami T., Yasuda Y. (2015). Terrestrial gamma radiation dose rate in Ryukyu Islands, subtropical region of Japan. Radiat. Prot. Dosim..

[35] Noguchi M. (1975). Liquid scintillation counting technique(X), special applications (2), measurements of radon activity. Radioisotopes.

[36] United Nations Scientific Committee on the Effects of Atomic Radiation (2000). Annex B: Exposures from natural radiation sources. Sources and Effects of Ionizing Radiation UNSCEAR 2000 Report to the General Assembly, with Scientific Annexes.

[37] Shiroma Y., Hosoda M., Ishikawa T., Sahoo S.K., Tokonami S., Furukawa M. (2015). Estimation of radon emanation coefficient for representative soils in Okinawa Japan. Radiat. Prot. Dosim..

[38] Nakaya S., Yasumoto J., Ha M.P., Aoki H., Kohara F., Masuda H., Masuoka K. (2018). Hydrochemical behavior of an underground dammed limestone aquifer in the subtropics. Hydrol. Process..

